# Metabolic Engineering of *Escherichia coli* for Production of Polyhydroxyalkanoates with Hydroxyvaleric Acid Derived from Levulinic Acid

**DOI:** 10.4014/jmb.2108.08016

**Published:** 2021-10-14

**Authors:** Doyun Kim, Sung Kuk Lee

**Affiliations:** 1 Department of Biomedical Engineering, Ulsan National Institute of Science and Technology (UNIST), Ulsan 44919, Republic of Korea; 2 Department of Chemical Engineering, Ulsan National Institute of Science and Technology (UNIST), Ulsan 44919, Republic of Korea; 3 Department of Energy Engineering, Ulsan National Institute of Science and Technology (UNIST), Ulsan 44919, Republic of Korea

**Keywords:** Levulinic acid, *Escherichia coli*, short-chain-length polyhydroxyalkanoates (scl-PHAs)

## Abstract

Polyhydroxyalkanoates (PHAs) are emerging as alternatives to plastics by replacing fossil fuels with renewable raw substrates. Herein, we present the construction of engineered *Escherichia coli* strains to produce short-chain-length PHAs (scl-PHAs), including the monomers 4-hydroxyvalerate (4HV) and 3-hydroxyvalerate (3HV) produced from levulinic acid (LA). First, an *E. coli* strain expressing genes (*lvaEDABC*) from the LA metabolic pathway of *Pseudomonas putida* KT2440 was constructed to generate 4HV-CoA and 3HV-CoA. Second, both PhaAB enzymes from *Cupriavidus necator* H16 were expressed to supply 3-hydroxybutyrate (3HB)-CoA from acetyl-CoA. Finally, PHA synthase (PhaC_Cv_) from *Chromobacterium violaceum* was introduced for the subsequent polymerization of these three monomers. The resulting *E. coli* strains produced four PHAs (w/w% of dry cell weight): 9.1 wt% P(4HV), 1.7 wt% P(3HV-*co*-4HV), 24.2 wt% P(3HB-*co*-4HV), and 35.6 wt% P(3HB-*co*-3HV-*co*-4HV).

## Introduction

Since 2019, the world has been dealing with the effects of the coronavirus pandemic (COVID-19) [1], which has caused wide-ranging environmental and health issues since it was officially classified as a global problem in 2020. One of the serious environmental impacts of the pandemic has been the sudden increase in demand for plastic products known as personal protective equipment (PPE) [2].

Bio-based plastics have emerged as a sustainable alternative to conventional plastics, and can help replace fossil fuels with renewable resources in the short term [3]. The use of bio-based plastics can reduce their carbon footprint, and biodegradable plastics increase the efficiency of plastic recycling and waste management, thereby lessening the environmental impact of plastic waste [4, 5]. Polyhydroxyalkanoates (PHAs) are highly appealing polymers derived from biomass and used as biodegradable plastics [6]. Among them, poly-3-hydroxybutyrate [P(3HB)] has been researched most.

P(3HB) is a 3-hydroxybutyrate (3HB)-based short-chain-length PHA (scl-PHA) with physical properties similar to those of petroleum-based plastics, including polypropylene and polystyrene [7]. However, P(3HB) is difficult to manufacture because of its high brittleness and high melting point, both limiting factors in its production and application [8, 9]. Glass and melting transition temperatures are important parameters related to the application of PHA [10]. To solve this problem, studies have been conducted to control PHA composition, and it has been confirmed that the properties of PHA can be improved by altering its composition [11]. Incorporating 4-hydroxyvalerate (4HV) or 3-hydroxyvalerate (3HV) monomers into PHAs has been shown to lower the melting point and improve flexibility without affecting the plastic decomposition efficiency [11-13]. However, the production of such copolymer PHAs has been limited owing to the high toxicity of petroleum-derived precursors like γ-valerolactone (GVL), valeric acid, or propionate, and the high cost of biological production of 4HV and 3HV monomers [8, 14, 15].

Renewable carbon sources are economically reasonable and have excellent potential as feedstock for industrial PHA production [10]. Levulinic acid (LA) is considered a 3HV and 4HV precursor for PHA production [16-18]. In addition, LA can be produced on industrial scale from cellulosic biomass at a cost as low as $0.04–$0.10/lb [19]. An engineered *Pseudomonas putida* strain showed potential for effective PHA production from LA using its LA metabolic pathway [18]. However, use of this *Pseudomonas* strain is hindered by incomplete knowledge of genetic information and non-availability of genetic manipulation tools when using low-cost carbon sources.

Biological PHA production studies should aim to improve the cost and efficiency of the process. Most natural microorganisms can easily synthesize PHA from various monomers, but only on laboratory-scale and from structurally related precursors [20]. PHA biosynthesis in hosts that do not naturally produce PHA may improve the quality and quantity of PHA [21]. In addition, the PHA synthesis pathway can also be modified to extend the range of products and control the monomer content [21]. *Escherichia coli* generally does not produce PHA, but its short doubling time and the comprehensive knowledge of its molecular genetics and physiology have made *E. coli* a pioneering organism in the study of PHA biosynthesis [22-25].

In this study, we investigated the biosynthesis of different forms of PHA in *E. coli* engineered with the LA catabolic pathway (*lva* pathway) derived from *P. putida* KT2440 [26]. The present study also proposes an alternative method to effectively supply HV monomers for PHA synthesis compared to using petroleum-derived precursors (GVL, valeric acid, or propionate).

## Materials and Methods

### Construction of Bacterial Strains and Plasmids

Table 1 lists the strains and plasmids used in this research. *E. coli* DH10B was used for all molecular cloning experiments. It was used as the parent strain of *E. coli* MG1655 developed for PHA production. *P. putida* KT2440 (DSMZ 6125) was procured from the German Collection of Microorganisms and Cell Cultures GmbH (DSMZ, Germany). *Cupriavidus necator* H16 (KCTC 22469) and *Chromobacterium violaceum* (KCTC 2897) were purchased from the Korean Collection for Type Cultures (KCTC, Korea).

Q5 High-Fidelity DNA polymerase, Taq DNA ligase, T4 polynucleotide kinase, and T5 exonuclease were procured from New England Biolabs (USA) and used for PCR and plasmid construction. All plasmids used in this study were constructed using the Biobrick plasmid [27]. To create pBbB6a-*lva*ED, pBbE6k-*lva*ABC, pBbA2c-*pha*C, and pBbA2c-*pha*CAB plasmids, the genes *lvaEDABC* of *P. putida* KT2440, phaC of *C. violaceum*, and *phaAB* of *C. necator* were amplified individually and cloned into each plasmid [28]. The RBS sequence was newly synthesized and applied to each gene using the Salis Lab RBS calculator. (Version 2.1) [29]. The plasmid was then transformed into *E. coli* using a MicroPulser electroporator (Bio-Rad, USA).

### Media and Cultivation Conditions

LA solution (Sigma-Aldrich, USA) was neutralized to pH 7 with NaOH and used for cultivation. For the gas chromatography–mass spectrometry (GC-MS) analysis, methyl benzoate (Acros Organics, USA), GVL, and PHA polymer granules (88 mol% 3HB, 12 mol% 3HV) (Sigma-Aldrich) were used. GVL was saponified with NaOH to prepare 4-hydroxyvaleric acid for GC-MS [30].

The recombinant *E. coli* strains were inoculated in 5 ml lysogeny broth (LB) (5 g/l yeast extract, 10 g/l tryptone, and 10 g/l NaCl) and cultured at 37°C for 10 h with 200 rpm shaking. For PHA production, the grown seed culture was transferred (1:40) into 40 ml MR medium (pH 7) containing: 4g/l (NH_4_)_2_HPO_4_, 6.67g/l KH_2_PO_4_, 0.8 g/l citric acid, 0.8g/l MgSO_4_·7H_2_O, 1.2 ml trace element solution (0.3 g/l CoCl_2_·H_2_O, 2.4 g/l FeCl_3_·6H_2_O, 0.3 g/l ZnCl_2_, 0.15 g/l CuCl_2_·2H_2_O, 0.075 g/l H_3_BO_3_, 0.3 g/l Na_2_MO_4_·2H_2_O, and 0.495 g/l MnCl_2_·4H_2_O), and 2.3 g/l LA (pH 7), with 15 g/l glucose. The bacteria were cultured in a 250-ml shake flask at 30°C for 96 h with 200 rpm shaking. When the optical density (OD_600_) reached 0.3, 0.1 mM isopropyl-D-1-thiogalactopyranoside (IPTG) and 50 nM tetracycline were added to induce gene expression. The medium was supplemented with ampicillin (100 μg/l), chloramphenicol (30 μg/l), and/or kanamycin (50 μg/l) depending on the resistance marker of the plasmids. A spectrophotometer was used to measure the OD_600_ (Libra S22; Biochrom, UK).

### PHA Analytical Methods

For the PHA study, 10 ml of culture was centrifuged for 20 min at 4°C at 2,600 ×*g* (Swingout rotor, Combi-514R, Hanil Scientific, Korea). The harvested cells were washed twice with triple-distilled water and the pellet was frozen at -80°C using a 2.5-L benchtop freeze dryer (Labconco, USA). PHA content and composition were analyzed by GC-MS using the following methanolysis method [31]: the dried cells were treated with a mixture of 1 ml chloroform, 1 ml of 15% (v/v) methanol, and 85% (v/v) sulfuric acid in 8 ml glass vials (WH224704, Wheaton, USA), and reacted in a dry bath (MaXtable H10, DAIHAN, Korea) at 100°C for 3 h. A polytetrafluoroethylene-lined cap was used to close the vials (WH240409, Wheaton, Millville, New Jersey, USA). The vials were cooled to room temperature after the reaction, and 1 ml of a 0.5% (v/v) methyl benzoate-chloroform solution and 1 ml triple distilled water were applied for phase acceleration and vortexed for 30 s. The phase separation was performed at room temperature (20~22°C) for 3 h. The sample (500 μl) obtained in the organic solvent layer was used for GC-MS analysis.

The analysis was performed on a 7890 B GC-MS (Agilent Technologies) system equipped with an Agilent J&W CycloSil-B column (113-6632). Helium was used at a flow rate of 1.5 ml/min, and 2 μl of the sample was injected. From a starting temperature of 60°C, the oven temperature was raised at the rate of 10°C/min for 7 min, and then increased at the rate of 30°C/min until reaching 250°C. All experiments were performed in triplicate.

## Results and Discussion

### Construction of Engineered *E. coli* for PHA Production

P(3HB) is the most widespread and best-characterized form of PHA and is produced by various bacterial species such as *Cupriavidus necator* [1], *Alcaligenes latus* [2], *Bacillus* spp. [4], *Azotobacter vinelandii* [3], and *Pseudomonas* sp. [4]. The incorporation of other monomer units into the 3HB polymer chains can result in copolymers with improved properties. Poly(3-hydroxybutyrate-*co*-3-hydroxyvalerate) is among the most popular copolymers, containing 3HV produced from propionic acid, propanol, valeric acid, pentanol, or heptanoic acid.

**Fig. 1:**
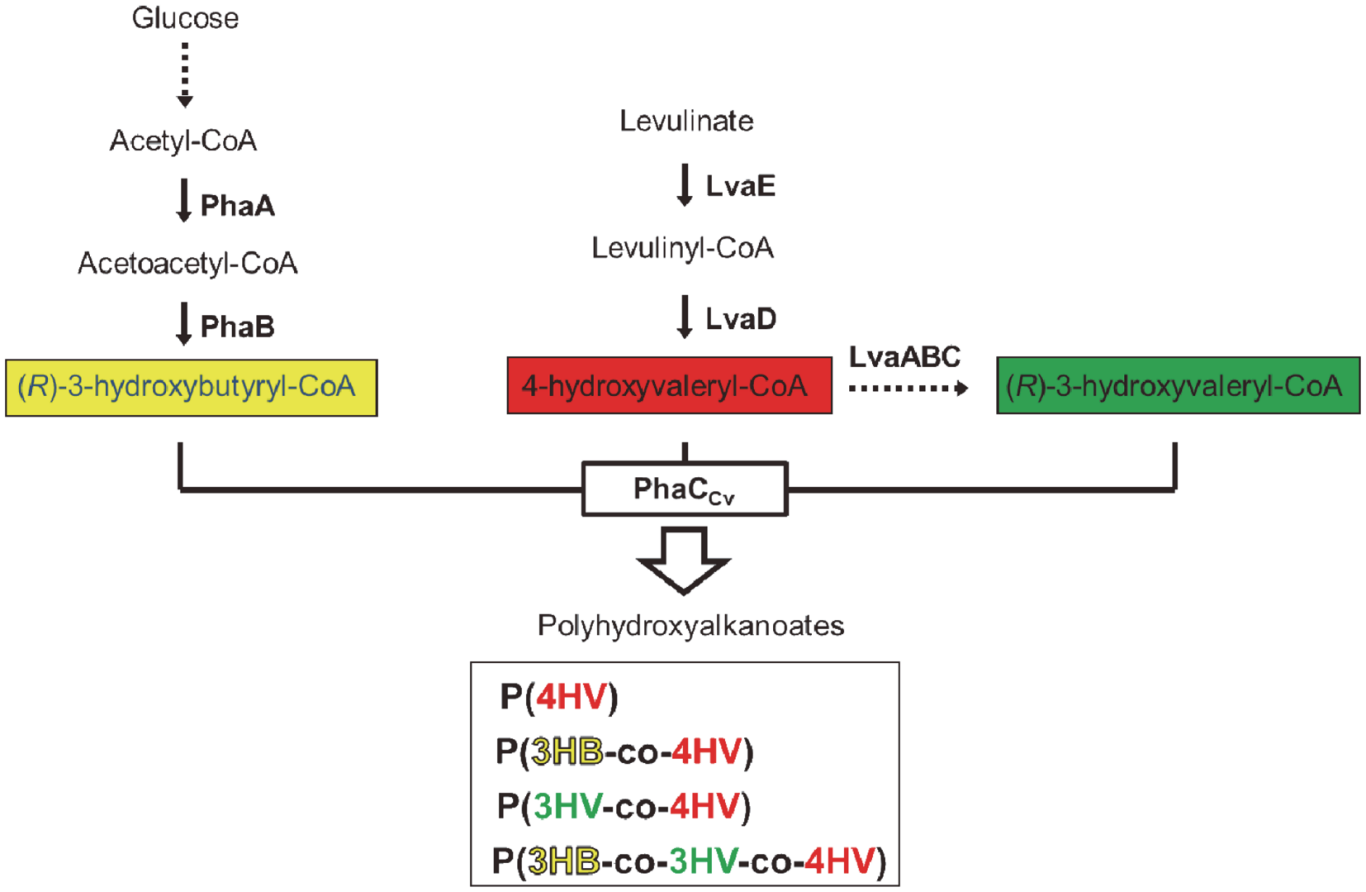
Metabolic pathways involved in the production of scl-PHA in this study. Enzymes: PhaA, acetyl-CoA acetyltransferase from *Cupriavidus necator* H16; PhaB, acetoacetyl-CoA reductase from *C. necator* H16; LvaA, 4- hydroxypentanoyl-CoA kinase; LvaB, 4-hydroxypentanoyl-CoA kinase; LvaC, 4-(phosphooxy)pentanoyl-CoA phosphatase/ mutase; LvaD, 4-oxopentanoyl-CoA 4-dehydrogenase; LvaE, short-chain acyl-CoA synthetase; PhaC_Cv_, poly(R)-3- hydroxyalkanoate polymerase from *Chromobacterium violaceum*.

Recently, a metabolic pathway in the *lva* operon that can synthesize 4HV-CoA and 3HV-CoA from LA, which can be cost-effectively produced from renewable cellulosic biomass, was identified in *P. putida* and characterized [26]. To synthesize HV monomer units from LA in *E. coli*, *P. putida* *lva*ED and *lva*ABC were cloned into pBbE6a-*gfp* and pBbE6k-*rfp* by replacing *gfp* and *rfp*, respectively, generating pBbB6a-*lva*ED and pBbE6k-*lva*ABC plasmids. pBbB6a-*lva*ED was introduced into *E. coli* to produce 4HV-CoA and pBbE6k-*lva*ABC to produce 3HV-CoA (Fig. 1).

*C. necator* H16 is a model microorganism for PHB production using the *phaABC* operon. To synthesize 3HB monomer units from glucose in *E. coli*, the *phaAB* genes encoding acetyl-CoA reductase and acetyl-CoA acetyltransferase from *C. necator* were cloned into pBbA2c-*rfp* together with PHA synthase (PhaC_Cv_) from *C. violaceum* under the tetracycline-inducible promoter (*tetR*-P_tetA_) to generate pBbA2c-*pha*CAB. To produce PHAs with HVs in *E. coli*, PHA synthase (PhaC_Cv_) from *C. violaceum* was expressed on the pBbA2 plasmid under the tetracycline-inducible promoter (tetR-P_tetA_) with and without *C. necator* *phaAB*, generating pBbA2c-*pha*CAB and pBbA2c-*pha*C plasmids, respectively.

### Poly-4-Hydroxyvalerate (p(4HV)) Production

Although *E. coli* does not naturally produce P(3HB), recombinant *E. coli* strains harboring the *C. necator* PHA biosynthesis genes have been used to efficiently produce P(3HB) mostly from glucose [32].

To investigate the possibility of high P(4HV) production from LA, a promising platform chemical that can be obtained from biomass, we constructed *E. coli* MG1655 harboring pBbB6a-*lva*ED and pBbA2c-*pha*C. In this system, only two enzymatic reactions are involved in the production of 4HV-CoA from LA, which is then polymerized by PhaC_Cv_ to form P(4HV), compared with the 12 enzymatic steps required for 3HB-CoA synthesis from glucose for P(3HB) production [33]. Approximately 1.6 g/l dry cell weight (DCW) with 9.0 wt% P(4HV) content was obtained from 2.3 g/l LA after 96 h cultivation of the PHV01 strain (Table 2). Even without the expression of PhaAB, trace amounts of 3HB (approximately 0.1 wt%) were incorporated into the PHA backbone. This might be due to 3-hydroxyacyl-CoA epimerase (FadB, FadJ) activity in the cell that converts (*S*)-3-hydroxybutyl-CoA released during beta-oxidation to (*R*)-3-hydroxybutyryl-CoA [34].

**Fig. 2:**
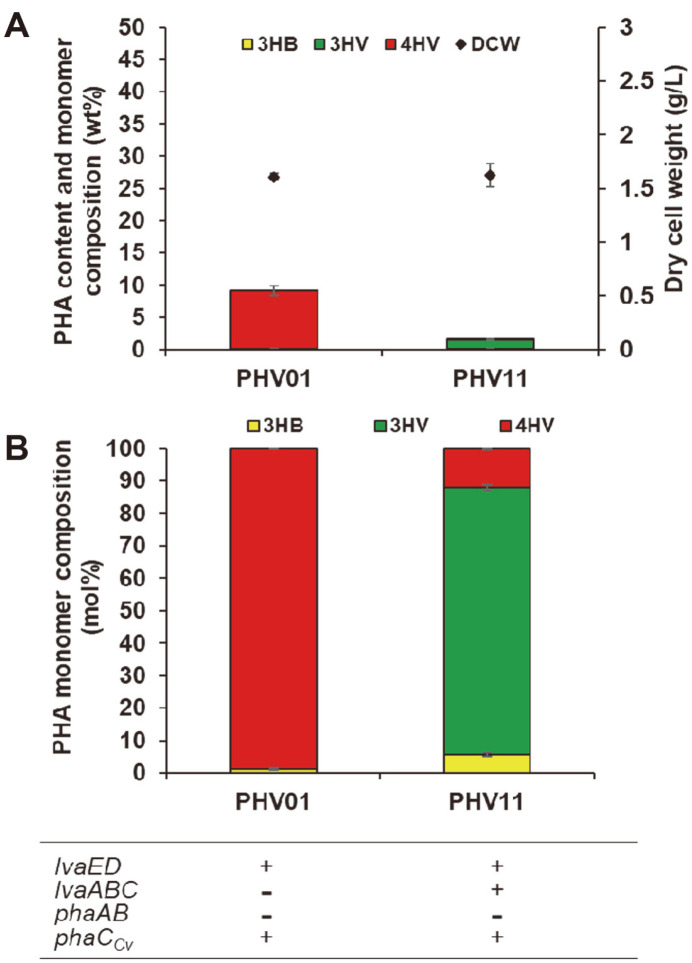
Cellular PHA content (wt%), dry cell weight (g/l) (A) and PHA monomer composition (mol%) (B) in PHV01 strain harboring pBbB6a-*lva*ED and pBbA2c-*pha*C, and PHV11 strain harboring pBbB6a-*lva*ED, pBbE6k-*lva*ABC and pBbA2c-*pha*C. Samples were taken and analyzed after 96 h of cultivation. Yellow, 3HB; red, 4HV; green, 3HV. The table shows the names of plasmid-expressed enzymes. Error bars represent the standard deviations of three independent cultivations.

In previous P(4HV) production studies, *P. putida* KT2440 with *lvaAB* deletion accumulated only a small amount (2 wt% cell content) of the P(4HV) homopolymer from 7.5 g/l LA [18] and an engineered *E. coli* strain also produced a small amount (1 wt%) of P(4HV) from 4HV as a substrate [35]. In this study, a higher (9.0 wt%) P(4HV) content was produced than those in previous studies; nevertheless, this amount is still low compared with the P(3HB) production of up to 80 wt% (Fig. 2) [36]. Considering a previous study that showed production of 100 g/l and 4.2 g/l/h of 4HV from LA in *E. coli* MG1655 [37], the low production of P(4HV) in the present study may be due to the low activity of the PHA synthase PhaC_Cv_ for 4HV-CoA, an unnatural substrate, and because the availability of 4HV-CoA for the synthesis of P(4HV) was not optimized. This study suggests that enzyme and metabolic engineering must be further explored to increase high P(4HV) production.

### Poly-3-Hydroxyvalerate-*co*-4-Hydroxyvalerate [P(3HV-*co*-4HV)] Production

P(4HV) production in microorganisms was still low (9.0 wt%), possibly owing to the low substrate specificity of the PHA synthase for 4HV-CoA. Because 4HV-CoA and 3HV-CoA could be generated from LA via the *lva* operon, we attempted to produce a copolymer. The PhaC_Cv_ of *C. violaceum* accumulates polymers comprising predominantly 3HV [38]. Therefore, the production of P(3HV-*co*-4HV) copolymer was investigated in the PHV11 strain by expressing the polymerase together with LvaED and LvaABC.

After 96 h of cultivation, a DCW of 1.6 g/l and P(3HV-*co*-4HV) copolymer with a molar ratio of 82.4 mol% of 3HV and trace amounts of 4HV were produced. The highest PHA concentration was determined to be 27.2 mg/l, which corresponds to a PHA content of 1.7 wt% of DCW. In contrast to the PHV01 expressing only LvaED, the molar proportion of 3HV in the copolymer rose to 82.4 mol% (Fig. 2). In a previous study, a molar ratio of 61.6 mol% 3HV and 37.8 mol% 4HV was achieved in *P. putida* KT2440 with the same PhaC_Cv_ enzyme but no PhaAB expression [18]. *P. putida* KT2440 produced 37.4 wt% P(3HV-*co*-4HV), which was approximately 22 times higher than that produced by the *E. coli* MG1655 strain.

The PHA monomer composition depends on the amount and proportion of monomers that are fed into the culture medium [39]. In the *P. putida* KT2440 study, LA was used at a high concentration of 7.5 g/l to induce nitrogen limitation [18], whereas 2.3 g/l (20 mM) LA was used in this study with *E. coli* MG1655. *P. putida* KT2440 probably produced a higher 4HV monomer proportion than *E. coli* MG1655 owing to the difference in LA concentration. For instance, the molar ratios of 3HB, 3HV, and 4HV were controlled by modulating the substrate concentration in a study on the production of P(3HB-*co*-4HV-*co*-3HV) using *Cupriavidus* sp. USMAA2-4 strain [8]. In another study using the *Cupriavidus* sp. L7L strain, the monomer proportion of 3HV and cellular PHA content was altered when the quantity of LA in the medium was varied (0.2–1.7%) [11]. In the present study, only one LA concentration was applied to evaluate the feasibility of PHA production in engineered *E. coli*. Additional metabolic engineering and LA feeding control can be performed to vary the monomer composition of PHAs. The production of PHA with higher 4HV proportion by *P. putida* can also be attributed to the higher rate of LA consumption by *P. putida* than the engineered *E. coli* PHV11 strain. Improving LA consumption in *E. coli* through further metabolic engineering can be expected to facilitate higher production of P(3HV-*co*-4HV).

### Poly-3-Hydroxybutyrate-*co*-4-Hydroxyvalerate [P(3HB-*co*-4HV)] Production

The inclusion of 4HV has been proven to improve the physical properties of commercialized PHB [30]. Although petroleum-derived precursors (GVL, valeric acid, or propionate) have been used to supply 4HV, LA, which is easily obtained from biomass, offers economic advantages as a substrate for 4HV synthesis. We therefore investigated the production of poly(hydroxybutyrate-*co*-hydroxyvalerate) (PHBV) copolymer from glucose and LA in engineered *E. coli* PHBV01.

**Fig. 3:**
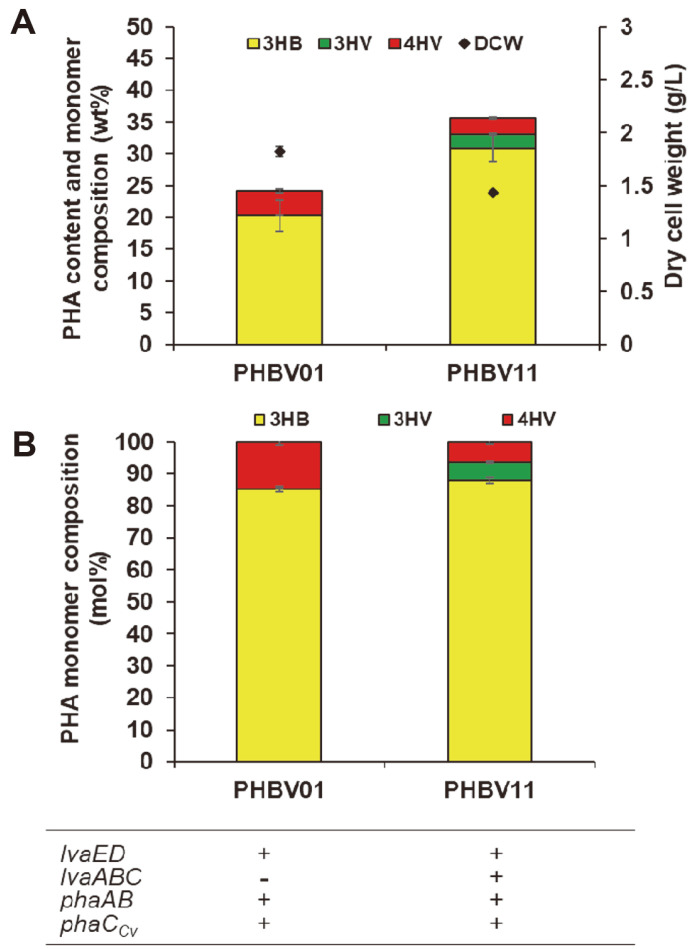
Cellular PHA content (wt%), dry cell weight (g/l) (A) and PHA monomer composition (mol%) (B) in PHBV01 harboring pBbB6a-*lva*ED and pBbA2c-*pha*CAB, and PHBV11 harboring pBbB6a-*lva*ED, pBbE6k-*lva*ABC and pBbA2c-*pha*CAB. Samples were taken and analyzed after 96 h of cultivation. Yellow, 3HB; red, 4HV; green, 3HV. The table indicates the enzymes overexpressed from plasmids. Error bars indicate standard deviations of three independent cultivations.

When *E. coli* PHBV01 expressing LvaED, PhaAB, and PhaC_Cv_ was grown in MR minimal medium with LA (20 mM) and glucose (15 g/l), 1.8 g/l DCW and 435.6 mg/l PHA titer were obtained after 96 h cultivation. The cellular PHA content and 4HV molar ratio were 24.2 wt% and 14.6 mol%, respectively (Table 2, Fig. 3). The molar fraction of the non-conventional monomer 4HV was not very low, even in the presence of the natural monomer 3HB of PHA synthase. However, the amount of 4HV decreased from 9.0 wt% to 3.9 wt% compared to that of the PHV01 strain without PhaAB expression, indicating that the PHA synthase exhibits higher substrate specificity for 3-hydroxyacyl-CoA (3HB-CoA) than 4-hydroxyacyl-CoA (4HV-CoA).

These results showed that the PHBV copolymer could be produced in a non-natural PHA-producing *E. coli* strain by introducing heterologous genes. The monomer molar ratio may be varied by controlling the monomer production rate. To increase both PHBV production and 4HV molar ratio, it is necessary to further engineer PHA synthase to increase its specificity for the non-natural substrate 4HV over 3HB.

### Poly-3-Hydroxybutyrate-*co*-3-Hydroxyvalerate-*co*-4-Hydroxyvalerate [P(3HB-*co*-3HV-*co*-4HV)] Production

Various plastics with specialized properties are required for specific applications. The properties of plastics can be controlled by polymer blending or the production of block copolymers with various monomers. We investigated the production of a random copolymer containing two 3-hydroxyacids (3HB and 3HV) and a 4-hydroxyacid (4HV) by supplying 3HB from glucose through PhaAB reactions and 3HV and 4HV from LA through LvaEDABC reactions.

After 96 h of cultivation, a P(3HB-*co*-3HV-*co*-4HV) terpolymer with 1.4 g/l DCW and 35.6 wt% and a monomer molar ratio of 88:6:6 were obtained from the PHBV11 strain. The content of 3HB monomer increased from 85.3 mol% in the terpolymer compared to 87.9 mol% in the PHBV produced by the PHV11 strain. However, the amount of total HV monomer was still 12.1 mol%. The DCW also reduced slightly from 1.6 g/l to 1.4 g/l (Table 2, Fig. 3). In comparison with *P. putida* KT2440 strains that produced PHBV with 9.4 mol% 3HB, 67.1 mol% 3HV, and 23.5 mol% 4HV [18], the *E. coli* strain produced PHBV with a higher proportion of the 3HB monomer.

The observed variation in terpolymer monomer ratio might be attributed to differences in PhaC_Cv_ expression, 3HB monomer availability under different PhaAB expression patterns, and variation in the cellular physiology of host cells *E. coli* MG1655 and *P. putida* KT2440.

In conclusion, *E. coli* provides a well-established culture strategy for achieving high cell densities and fragility of cells, facilitating the simple isolation and purification of biopolymers [20, 40]. Four distinct varieties of PHAs, namely p(4HV), p(3HV-*co*-4HV), p(3HB-*co*-4HV), and p(3HB-*co*-3HV-*co*-4HV) were produced using metabolically engineered *E. coli*. Although *E. coli* produces less PHA than *P. putida* KT2440, the results from this study demonstrate that it can serve as a host to produce PHAs with HV monomers synthesized from LA after further metabolic engineering. This research also provides an alternative approach for effectively supplying HV monomers from a cost-effective alternative for PHA synthesis rather than using costly substrates like GVL, valeric acid, or propionate.

## Figures and Tables

**Table 1:** Strains and plasmids.

Strains and plasmids	Genotype and description	Reference
Strains		
MG1655	*E. coli* K-12 F^–^λ^–^*ilvG*^–^*rfb*-50*rph-1*	[41]
DH10B	F^–^*mcrA* Δ(*mrr-hsdRMS-mcrBC*) *φ*80*lacZ*ΔM15 Δ*lacX74 recA1 endA1 araD*139 Δ(*ara-leu*)7697 *galU galK* λ^–^*rpsL*(Str^R^) *nupG*	[42]
PHV01	MG1655 harboring pBbB6a-*lva*ED, and pBbA2c-*pha*C	This study
PHV11	MG1655 harboring pBbB6a-*lva*ED, and pBbE6k-*lva*ABC, and pBbA2c-*pha*C	This study
PHBV01	MG1655 harboring pBbB6a-*lva*ED, and pBbA2c-*pha*CAB	This study
PHBV11	MG1655 harboring pBbB6a-*lva*ED, pBbE6k-*lva*ABC, and pBbA2c-*pha*CAB	This study
Plasmids		
pBbB6a-*gfp*	pBBR1 origin, carrying *gfp* under the P_LlacO-1_, Amp^R^	[27]
pBbE6k-*rfp*	ColE1 origin, carrying *rfp* under the P_LlacO-1_, Km^R^	[27]
pBbA2c-*rfp*	p15A origin, carrying rfp under the P_tetA_, Cm^R^	[27]
pBbB6a-*lvaED*	pBbB6a-*gfp* with Δ*gfp::lvaED* from *P. putida* KT2440, Amp^R^	This study
pBbE6k-*lvaABC*	pBbE6k-*rfp* with Δ*rfp::lvaABC* from *P. putida* KT2440, Km^R^	This study
pBbA2c-*phaC*	pBbA2c-*rfp* with Δ*rfp::phaC* from *C. violaceum*, Cm^R^	This study
pBbA2c-*phaCAB*	pBbA2c-*rfp* with Δ*rfp::phaCAB* from *C. violaceum*, and *C. necator* H16, Cm^R^	This study

**Table 2:** PHA content in the cell biomass (wt%), monomer molar ratio (mol%) and dried cell weight (g/l) of strains.

	PHV01	PHV11	PHBV01	PHBV11
3HB (mol%)	Tr^a^	Tr^a^	85.3 ± 1.0	87.9 ± 0.8
3HV (mol%)	ND^b^	82.4 ± 0.9	ND^b^	5.7 ± 0.2
4HV (mol%)	98.7 ± 0.1	Tr^a^	14.6 ± 1.0	6.4 ± 0.6
CDM (g/l)	1.6 ± 0.0	1.6 ± 0.1	1.8 ± 0.0	1.4 ± 0.0
PHA content (wt%)	9.1 ± 0.7	1.7 ± 0.1	24.2 ± 2.8	35.6 ± 2.2

^a^Tr, Detected in trace quantity ( < 0.5%, w/w)

^b^ND, Not detectable

## References

[1] Reinecke F, Steinbüchel A (2009). *Ralstonia eutropha* strain H16 as model organism for PHA metabolism and for biotechnological production of technically interesting biopolymers. Microb. Physiol..

[2] Yu J (2007). Microbial production of bioplastics from renewable resources. Bioprocessing for value-added products from renewable resources.

[3] Pettinari MJ, Vázquez GJ, Silberschmidt D, Rehm B, Steinbüchel A, Méndez BS (2001). Poly (3-hydroxybutyrate) synthesis genes in Azotobacter sp. strain FA8. Appl. Environ. Microbiol..

[4] Lütke‐Eversloh T, Steinbüchel A (2004). Microbial polythioesters. Macromol. Biosci..

[5] Napper IE, Thompson RC (2019). Environmental deterioration of biodegradable, oxo-biodegradable, compostable, and conventional plastic carrier bags in the sea, soil, and open-air over a 3-year period. Environ. Sci. Technol..

[6] Bhatia SK, Gurav R, Choi T-R, Jung H-R, Yang S-Y, Moon Y-M (2019). Bioconversion of plant biomass hydrolysate into bioplastic (polyhydroxyalkanoates) using *Ralstonia eutropha* 5119. Bioresour. Technol..

[7] Lemoigne M (1926). Products of dehydration and of polymerization of β-hydroxybutyric acid. Bull. Chem. Soc. Japan.

[8] Muzaiyanah AR, Amirul AA (2013). Studies on the microbial synthesis and characterization of polyhydroxyalkanoates containing 4-hydroxyvalerate using γ-valerolactone. Appl. Biochem. Biotechnol..

[9] Raza ZA, Abid S, Banat IM (2018). Polyhydroxyalkanoates: Characteristics, production, recent developments and applications. Int. Biodeterior. Biodegradation.

[10] Możejko-Ciesielska J, Kiewisz R (2016). Bacterial polyhydroxyalkanoates: Still fabulous?. Microbiol. Res..

[11] Sheu D-S, Chen Y-LL, Jhuang W-J, Chen H-Y, Jane W-N (2018). Cultivation temperature modulated the monomer composition and polymer properties of polyhydroxyalkanoate synthesized by *Cupriavidus* sp. L7L from levulinate as sole carbon source. Int. J. Biol. Macromol..

[12] Gahlawat G, Soni SK (2017). Valorization of waste glycerol for the production of poly (3-hydroxybutyrate) and poly (3-hydroxybutyrate-*co*-3-hydroxyvalerate) copolymer by *Cupriavidus necator* and extraction in a sustainable manner. Bioresour. Technol..

[13] Schmack G, Gorenflo V, Steinbüchel A (1998). Biotechnological production and characterization of polyesters containing 4-hydroxyvaleric acid and medium-chain-length hydroxyalkanoic acids. Macromolecules.

[14] Lee W-H, Loo C-Y, Nomura CT, Sudesh K (2008). Biosynthesis of polyhydroxyalkanoate copolymers from mixtures of plant oils and 3-hydroxyvalerate precursors. Bioresour. Technol..

[15] Valentin HE, Steinbüchel A (1995). Accumulation of poly(3-hydroxybutyric acid-*co*-3-hydroxyvaleric acid-*co*-4-hydroxyvaleric acid) by mutants and recombinant strains of *Alcaligenes eutrophus*. J. Environ. Polymer Degradation.

[16] Koller M, Hesse P, Fasl H, Stelzer F, Braunegg G (2017). Study on the effect of levulinic acid on whey-based biosynthesis of Poly(3-hydroxybutyrate-*co*-3-hydroxyvalerate) by *Hydrogenophaga pseudoflava*. Appl. Food Biotechnol..

[17] Novackova I, Kucera D, Porizka J, Pernicova I, Sedlacek P, Koller M (2019). Adaptation of *Cupriavidus necator* to levulinic acid for enhanced production of P(3HB-*co*-3HV) copolyesters. Biochem. Eng. J..

[18] Cha D, Ha HS, Lee SK (2020). Metabolic engineering of *Pseudomonas putida* for the production of various types of short-chain-length polyhydroxyalkanoates from levulinic acid. Bioresour. Technol..

[19] Bozell JJ, Moens L, Elliott DC, Wang Y, Neuenscwander GG, Fitzpatrick SW (2000). Production of levulinic acid and use as a platform chemical for derived products. Resour. Conserv. Recycl..

[20] Favaro L, Basaglia M, Casella S (2019). Improving polyhydroxyalkanoate production from inexpensive carbon sources by genetic approaches: a review. Biofuels Bioprod. Biorefining.

[21] Kidwell J, Valentin HE, Dennis D (1995). Regulated expression of the *Alcaligenes eutrophus* pha biosynthesis genes in *Escherichia coli*. Appl. Environ. Microbiol..

[22] Langenbach S, Rehm BHA, Steinbüchel A (1997). Functional expression of the PHA synthase gene *phaC1* from *Pseudomonas* aeruginosa in *Escherichia coli* results in poly(3-hydroxyalkanoate) synthesis. FEMS Microbiol. Lett..

[23] Ren Q, Sierro N, Kellerhals M, Kessler B, Witholt B (2000). Properties of engineered poly-3-hydroxyalkanoates produced in recombinant *Escherichia coli* strains. Appl. Environ. Microbiol..

[24] Ren Q, Beilen JBv, Sierro N, Zinn M, Kessler B, Witholt B (2005). Expression of PHA polymerase genes of *Pseudomonas putida* in *Escherichia coli* and its effect on PHA formation. Antonie Van Leeuwenhoek.

[25] Wang Q, Yu H, Xia Y, Kang Z, Qi Q (2009). Complete PHB mobilization in *Escherichia coli* enhances the stress tolerance: a potential biotechnological application. Microb. Cell Fact..

[26] Rand JM, Pisithkul T, Clark RL, Thiede JM, Mehrer CR, Agnew DE (2017). A metabolic pathway for catabolizing levulinic acid in bacteria. Nat. Microbiol..

[27] Lee TS, Krupa RA, Zhang F, Hajimorad M, Holtz WJ, Prasad N (2011). BglBrick vectors and datasheets: A synthetic biology platform for gene expression. J. Biol. Eng..

[28] Gibson DG, Young L, Chuang R-Y, Venter JC, Hutchison CA, Smith HO (2009). Enzymatic assembly of DNA molecules up to several hundred kilobases. Nat. Methods.

[29] Reis AC, Salis HM (2020). An automated model test system for systematic development and improvement of gene expression models. ACS Synth. Biol..

[30] Gorenflo V, Schmack G, Vogel R, Steinbüchel A (2001). Development of a process for the biotechnological large-scale production of 4-hydroxyvalerate-containing polyesters and characterization of their physical and mechanical properties. Biomacromolecules.

[31] Juengert JR, Bresan S, Jendrossek D (2018). Determination of polyhydroxybutyrate (PHB) content in *Ralstonia eutropha* using gas chromatography and nile red staining. Bio Protoc..

[32] Peña C, Castillo T, García A, Millán M, Segura D (2014). Biotechnological strategies to improve production of microbial poly‐(3‐hydroxybutyrate): a review of recent research work. Microb. Biotechnol..

[33] Matsumoto Ki, Yamada M, Leong CR, Jo S-J, Kuzuyama T, Taguchi S (2011). A new pathway for poly (3-hydroxybutyrate) production in *Escherichia coli* and *Corynebacterium glutamicum* by functional expression of a new acetoacetyl-coenzyme A synthase. Biosci. Biotechnol. Biochem..

[34] Snell KD, Feng F, Zhong L, Martin D, Madison LL (2002). YfcX enables medium-chain-length poly (3-hydroxyalkanoate) formation from fatty acids in recombinant *Escherichia coli*
*fadB* strains. J. Bacteriol..

[35] Liu S-J, Steinbüchel A (2000). A novel genetically engineered pathway for synthesis of poly(hydroxyalkanoic acids) in *Escherichia coli*. Appl. Environ. Microbiol..

[36] Kim BS, Lee SY (2000). Production of poly (3-hydroxybutyrate) from inexpensive substrates. Enzyme Microb. Technol..

[37] Kim D, Sathesh-Prabu C, JooYeon Y, Lee SK (2019). High-level production of 4-hydroxyvalerate from levulinic acid via whole-cell biotransformation decoupled from cell metabolism. J. Agric. Food Chem..

[38] Steinbüchel A, Debzi E-M, Marchessault RH, Timm A (1993). Synthesis and production of poly (3-hydroxyvaleric acid) homopolyester by *Chromobacterium violaceum*. Appl. Microbiol. Biotechnol..

[39] Tripathi L, Wu L-P, Dechuan M, Chen J, Wu Q, Chen G-Q (2013). *Pseudomonas putida* KT2442 as a platform for the biosynthesis of polyhydroxyalkanoates with adjustable monomer contents and compositions. Bioresour. Technol..

[40] Aldor IS, Keasling JD (2003). Process design for microbial plastic factories: metabolic engineering of polyhydroxyalkanoates. Curr. Opin. Biotechnol..

[41] Blattner FR, Plunkett G, Bloch CA, Perna NT, Burland V, Riley M (1997). The complete genome sequence of *Escherichia coli* K-12. Science.

[42] Durfee T, Nelson R, Baldwin S, Plunkett G, Burland V, Mau B (2008). The complete genome sequence of *Escherichia coli* DH10b: insights into the biology of a laboratory workhorse. J. Bacteriol..

